# Releasing Dynamic of Serum ST2 and Calprotectin in Patients with Acute Ischemic Stroke

**DOI:** 10.3390/diagnostics14131331

**Published:** 2024-06-23

**Authors:** Ana Sruk, Hrvoje Budinčević, Ana-Maria Šimundić, Lora Dukić, Tena Sučić Radovanović, Helena Čičak, Daria Pašalić

**Affiliations:** 1 Department of Neurology, Sveti Duh University Hospital, 10000 Zagreb, Croatia; srukana@gmail.com (A.S.); hbudincevic@gmail.com (H.B.); 2 Department of Neurology and Neurosurgery, Faculty of Medicine, J. J. Strossmayer University of Osijek, 31000 Osijek, Croatia; 3 Unit for Preanalytics, Department of Global Medical & Clinical Affairs, Business Greiner Bio-One GmbH, 4550 Kremsmünster, Austria; am.simundic@gmail.com; 4 Faculty of Pharmacy and Medical Biochemistry, Zagreb University, 10000 Zagreb, Croatia; 5 Department of Medical Laboratory Diagnostics, Sveti Duh University Hospital, 10000 Zagreb, Croatia; lora.dukic@gmail.com (L.D.); helena.cicak5@gmail.com (H.Č.); 6 Department of Radiology, Sveti Duh University Hospital, 10000 Zagreb, Croatia; tenas7@yahoo.co.uk; 7 Department of Medical Chemistry, Biochemistry and Clinical Chemistry, University of Zagreb School of Medicine, 10000 Zagreb, Croatia

**Keywords:** calprotectin, dynamics, ischemic stroke, outcome, prognostic accuracy, ST2

## Abstract

This study investigated the releasing dynamics of serum ST2 and calprotectin in patients with acute IS. The study included acute IS patients (N = 20) with an NIH Stroke Scale score ≥8. Sampling was performed at seven time points: after admission (T0) and at the following 24 h consecutive intervals (T1–T6). Primary outcome at 90 days was evaluated using the modified Rankin scale: 0–2 for good and 3–6 for poor functional outcome. The secondary outcome was all-cause mortality after 90 days. Fifteen patients had a poor outcome, and eight died. Results showed a statistically significant difference in ST2 concentrations between good and poor outcomes at T0 (*p* = 0.04), T1 (*p* = 0.006), T2 (*p* = 0.01), T3 (*p* = 0.021), T4 (*p* = 0.007), T5 (*p* = 0.032), and for calprotectin T6 (*p* = 0.034). Prognostic accuracy was highest for ST2 at T1 for a cut-off > 18.9 µg/L (sensitivity 80% and specificity 100.0%) and for calprotectin at T5 for a cut-off > 4.5 mg/L (sensitivity 64.3% and specificity 100.0%). Serum ST2 and calprotectin-releasing dynamics showed a valuable prognostic accuracy for IS outcomes.

## 1. Introduction

Ischemic stroke (IS) remains the most significant cause of disability and one of the most prevalent causes of mortality worldwide despite significant advancements in prevention and treatment [1]. It represents a severe public health problem with significant economic and social repercussions [2]. Providing an early prognosis of the IS outcome would be of great significance for patients and healthcare professionals [3]. Among clinical tools, the National Institute of Health Stroke Scale (NIHSS) demonstrated a prognostic value in the progression and outcome of IS [4,5]. Advanced neuroimaging techniques, such as perfusion imaging using computed tomography (CT) or magnetic resonance imaging (MRI), were shown to be crucial in predicting the response to therapy and influencing the outcome of IS [6]. These neuroimaging techniques are not always available and are not routinely performed. Therefore, efforts are required to find biomarkers that are simple to put into practice for IS outcome prediction. Considering many pathophysiological processes related to IS, serum biomarkers may be a valuable tool [7], including serum biomarkers for inflammation, coagulation, and heart damage [8,9,10]. The blood–brain barrier and the delayed response of the periphery, which do not promptly reflect the pathophysiological changes brought on by neuron injury, have prevented the identification of sensitive and specific biomarkers that would meet the criteria for broader application [11].

Increasing data suggest that inflammation plays a crucial role in causing tissue damage after a stroke [12]. In all types of strokes, inflammatory cells are present and active in both the brain tissue and the cerebrospinal fluid. The detrimental pro-inflammatory response has been associated with a number of cytokines and signaling pathways; however, little is known about the mediators that connect the initial vascular injury to the systemic immune response and the relationship between the reparative and pro-inflammatory immune responses. An interleukin signaling route strongly considered to play this role is the IL-33/ST2 axis [13]. ST2, also known as interleukin-1 receptor-like 1 (IL1RL1), is a protein receptor involved in immune responses and inflammation. The specific ST2 ligand is IL-33, a member of the class of Toll-like/IL-1 receptors. There are two types of ST2: soluble and transmembrane. Clinical investigations have demonstrated that increased serum concentrations of ST2 were associated with worse outcomes and higher mortality after IS [3,14], and they were also associated with a higher incidence of IS in a large cohort of participants followed for around 11 years [15]. Another inflammatory biomarker that has been studied more recently is calprotectin (S100A8/A9), a heterodimer composed of the S100 family of calcium-modulated proteins. Calprotectin belongs to the class of molecules known as danger-associated molecular patterns (DAMPs), which in part stimulate inflammation by activating TLR4 and RAGE, two receptors that are important to the onset and development of atherosclerosis [16,17]. As suggested by the possibility that calprotectin is a prognostic marker, high calprotectin concentrations have recently been found to be independently linked to a higher likelihood of poor clinical outcomes for IS [18,19,20]. 

The study’s design, patient demographics, sample size, biomarker measurement methodologies, and outcome measures are just a few examples of the diversity in the methods used in the previous studies looking at the role of ST2 and calprotectin in IS. Furthermore, the majority of the studies collected serum biomarkers at only one or a few specific time points. Understanding the dynamics of specific biomarker release is crucial for selecting an optimal time point for biomarker measurement. For this reason, we determined biomarker concentrations at consecutive time points to identify those with the highest prognostic accuracy. 

We hypothesized that earlier release of the biochemical markers ST2 and calprotectin into the circulation and higher serum concentrations would be associated with a worse outcome of the IS. This study aimed to investigate the prognostic accuracy of the dynamics of the release of the serum biomarkers ST2 and calprotectin in IS outcomes.

## 2. Materials and Methods

### 2.1. Study Design and Subjects

An observational, prospective study on prognostic accuracy was performed. The study was conducted at the Department of Neurology and the Department of Medical Laboratory Diagnostics of the Sveti Duh University Hospital. The research complies with all relevant national regulations and institutional policies, has been approved by the Ethical Board of the Sveti Duh University Hospital, and is registered on the ClinicalTrials.gov public registry (NCT04607031). The target population was patients with IS. The accessible population was patients with IS admitted to the Department of Neurology with a diagnosis of IS confirmed by two experienced neurologists. Based on previously published data for ST-2 between survivors and deceased (Dieplinger et al.’s research [3]), we calculated the number of required subjects with 80% power and α = 0.05. We decided to include 20 patients in the study.

Inclusion criteria were as follows: age ≥18 years, the onset of IS symptoms within 24 h, an initial NIHSS ≥ 8 [21], including patients with large vessel occlusion who were not transferred to other institutions for mechanical thrombectomy (MT) due to different reasons, and the consent of the patient, his or her legal representative, or guardian. Non-inclusion criteria were as follows: the unknown time of onset of symptoms; symptoms lasting longer than 24 h; patient conditions in which, according to the attending neurologist’s assessment, additional blood sampling could have an unfavorable effect on the outcome of the disease; the use of thrombolytic therapy and/or MT; and the presence of the following associated conditions: myocardial infarction, heart failure, malignant disease, immunological disease, severe infection, and pregnancy. Patients diagnosed with a transient ischemic attack, stroke mimics, patients urgently transferred to another institution for MT, and patients with IS caused by rare diseases were excluded from the study. 

Patient recruitment began on 7 September 2019, with the first sampling taking place on September 10th, marking the first patient to meet the inclusion criteria. The twentieth patient sample was taken on 22 December 2019, marking the end of patient recruitment. During the recruitment period, we immediately disqualified patients who met any of the exclusion criteria without performing any sampling for this study. 

Table 1 shows the procedure manual and breakdown of study time points in relation to time, blood sampling, and clinical and neurological indices, providing a comprehensive overview of the protocol’s execution and the corresponding assessments conducted throughout the study duration. At time points T0–T6, except for sampling for ST2 and calprotectin, we also performed routine laboratory tests aligned with standard practice for diagnosing and monitoring strokes. 

The primary outcome was the functional outcome according to the modified Rankin scale (mRS) after 90 days: 0–2 was defined as a good outcome and 3–6 as a poor outcome. The deceased are defined as mRS 6 [22]. The secondary outcome was all-cause mortality at 90 days. 

### 2.2. Blood Sampling

A nurse or technician sampled blood according to the EFML-COLABIOCLI guidelines [23]. Blood was collected into 6 mL serum tubes (Kima, Piove di Sacco, Italy) and 2 mL K2 ethylenediaminetetraacetic acid (EDTA) tubes (Kima, Piove di Sacco, Italy). The blood was sampled seven times immediately after admission to the Department of Neurology (T0) and at 7:30 a.m. every morning (first to sixth morning) after admission to the hospital, respectively, after the onset of the IS symptoms. These six sampling time points are defined in intervals of 24 h (T1–T6). Serum tubes were kept at room temperature for 30 min to allow them to clot, and immediately after, they were centrifuged at 3000× *g* for 10 min. After routine biochemistry analysis, serum was aliquoted into a minimum of three aliquots of 500 µL and stored at −20 °C to analyze the biochemical markers ST2 and calprotectin.

### 2.3. Methods

The leading investigator neurologist collected data from the hospital information system and performed neurological examinations daily during the patient’s hospitalization. The following data were collected or determined for each patient: sex, age, body mass, body height, body mass index (BMI), NIHSS, brain infarct volume, the exact time of onset of symptoms, smoking, excessive alcohol consumption, psychotropic substances abuse, arterial hypertension, atrial fibrillation, the presence of a heart valve, dyslipidemia, diabetes, coronary disease, liver dysfunction, renal dysfunction, peripheral arterial disease, previous stroke or transient ischemic attack, positive family history, IS complications, IS etiology according to the Trial of Org 10172 in Acute Stroke Treatment (TOAST) criteria [24], IS localization according to the Oxfordshire Community Stroke Project (OCSP) criteria [25], and prognostic indices the Prognostic Nutritional Index (PNI) and the Glasgow Prognostic Score (GPS).

### 2.4. Determination of Clinical Neurological Indices

The prognostic indices of PNI (a formula that includes albumin and leukocyte count) and GPS (a formula that includes CRP and albumin) were calculated. The size of the IS was analyzed based on the findings of brain CT and brain CT volumetry according to Pullica’s formula [26] in patients who underwent neuroradiological assessment that included brain CT initially and at an interval of at least 20–24 h. IS classification was performed using the TOAST [24] and OCSP criteria [25]. The severity of IS was clinically assessed using the NIHSS at each time point. Stroke severity is categorized as follows: no stroke symptoms, 0; minor stroke, 1–4; moderate stroke, 5–15; moderate to severe stroke, 16–20; and severe stroke, 21–42 [27]. Stroke outcome was assessed using the mRS at days 6 and 90 (± 7 days), with a comparison of disability using the same rating scale for the subject’s condition before hospitalization [22].

### 2.5. Determination of Hematological and Biochemical Parameters

The ST2 concentrations were measured by the enzyme-linked immunosorbent assay method using the Quantikine ELISA Human ST2/IL-33 R Immunoassay (R&D Systems, Minneapolis, MN, USA) on the automated ELISA analyzer ThunderBolt (Gold Standard Diagnostics, Davis, CA, USA). The manufacturer performed the precision of the ELISA kit for estimating ST2 at three concentration levels. The CV%s of intra-assay precision for the following concentration levels (273 pg/mL, 628 pg/mL, and 1027 pg/mL) were 5.6%, 4.4%, and 4.5%, respectively.

The calprotectin concentrations were measured by turbidimetric assay Buhlmann fCAL turbo (Buhlmann Laboratories AG, Schonenbuch, Switzerland) on an analyzer Atellica Solution (Siemens Health, Erlangen, Germany). Precision verification was performed for the fCAL turbo reagent on two patients’ samples with high (3.9 mg/L) and low (2.9 mg/L) concentrations. The estimated CV% for both concentration levels were 7.1% and 8.3%, respectively. The acceptance criteria were defined precision declared by the manufacturer, which was 9.1% [28].

### 2.6. Statistical Analysis

Considering the number of included patients, non-parametric statistical tests were applied. Categorical data were expressed as an absolute number and ratio, while continuous data were expressed as the median and interquartile range (IQR). Friedman ANOVA tested the difference between concentrations at different time points from the same patient (paired variables). The post hoc test was used for the pairwise comparison of variables, according to Conover, 1999.

The difference between the two groups of unpaired variables (good and poor outcomes) at the same point was tested by the Mann–Whitney test. The association of different parameters was tested by performing Spearman’s rank correlation.

Analysis of prognostic accuracy included sensitivity, specificity, overall accuracy (area under the ROC curve), and positive and negative likelihood ratios. Multivariate logistic regression was used to identify statistically significant predictors of outcome. The significance level was *p* < 0.05, and all confidence intervals were provided at 95%. Statistical analysis was performed using MedCalc Statistical Software (Version 20.027 – 64-bit, Ostend, Belgium; https://www.medcalc.org (accessed on 14 January 2022)).

## 3. Results

We studied 20 patients with IS (median age 84 years, 0.65 women). Table 2 presents the baseline characteristics of patients with IS, providing a comprehensive overview of demographic information, clinical parameters, and relevant medical history at the onset of the study. 

In terms of the primary functional outcome after a 90-day period, a total of fifteen patients had poor outcomes, as indicated by mRS 3–6. Eight patients died in relation to the secondary outcome, which was represented as all-cause mortality at 90 days (Table 3). 

Table 4 displays the median concentrations of ST2 and calprotectin at measurement time points T0–T6, expressed in median hours, offering insight into the temporal dynamics of these biomarkers throughout the study duration.

Results regarding stroke outcome and survival are represented in Table 5 and Table 6, respectively. We determined statistically significant difference in ST2 concentrations between good and poor outcomes at T0 (*p* = 0.04), T1 (*p* = 0.006), T2 (*p* = 0.01), T3 (*p* = 0.021), T4 (*p* = 0.007), and T5 (*p* = 0.032) time point (Table 5). The difference in ST2 concentrations was also found between survivors and deceased for the T1 (*p* = 0.014), T2 (*p* = 0.009), T3 (*p* = 0.003), T4 (*p* = 0.004), T5 (*p* = 0.041), and T6 (*p* = 0.027) (Table 6). Prognostic accuracy was highest for ST2 at T1 for a cut-off >18.9 µg/L (sensitivity 80% and specificity 100%) (Table 7). Table 7 presents the prognostic accuracy of ST2 concentrations at time points T0 through T6, providing a detailed analysis of their prognostic accuracy across various stages of the study period. 

**Figure 1:**
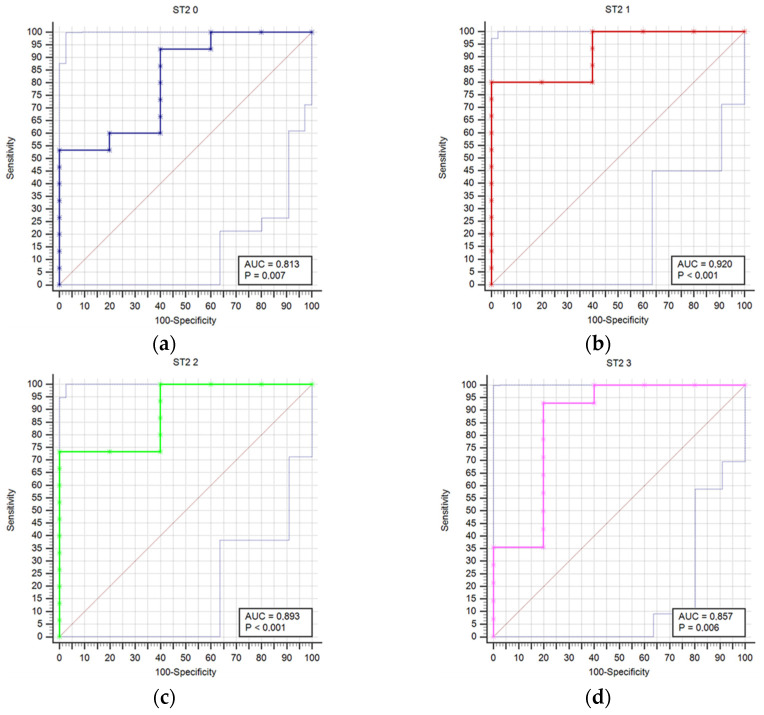

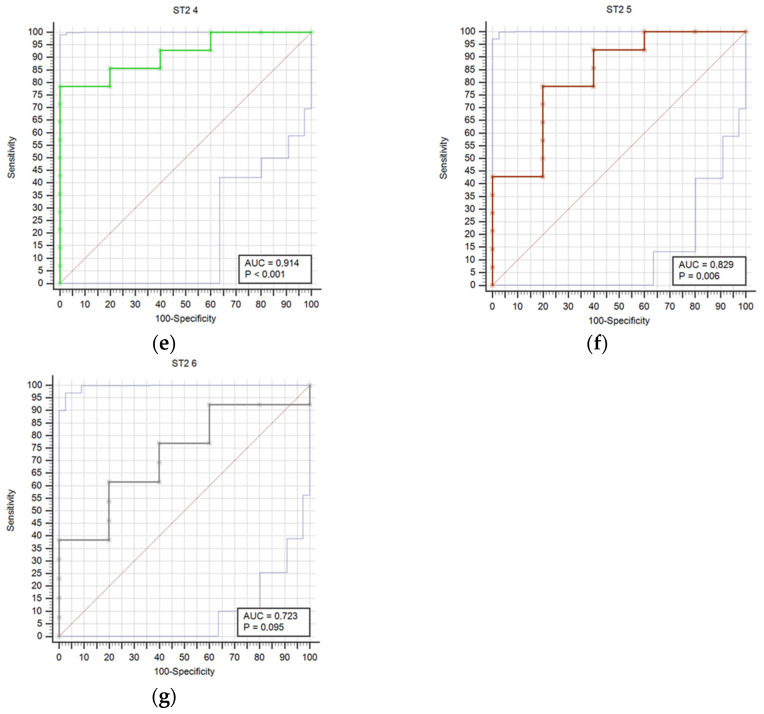
Receiver operating characteristic curves (ROC) comparison for outcome prediction based on the serum ST concentrations at time points 0 (**a**), 1 (**b**), 2 (**c**), 3 (**d**), 4 (**e**), 5 (**f**), and 6 (**g**).

Figure 1 compares receiver operating characteristic curves (ROC) for outcome prediction based on serum ST2 concentrations at time points T0 through T6, providing a visual representation of ST2’s predictive performance across various stages of the study.

Results regarding the association of stroke outcome and survival with calprotectin concentrations are represented in Table 8 and Table 9, respectively. We determined a statistically significant difference in calprotectin concentrations between good and poor outcomes at the T6 (*p* = 0.034) time point (Table 8). The difference in calprotectin concentrations was also found between survivors and deceased for the T5 (*p* = 0.004) time point (Table 9). Prognostic accuracy was highest for calprotectin at the T5 time point for a cut-off > 4.5 mg/L (sensitivity 64.3% and specificity 100.0%) (Table 10). Table 10 provides a thorough assessment of the prognostic accuracy of calprotectin concentrations at consecutive intervals during the study period (T0–T6).

**Figure 2:**
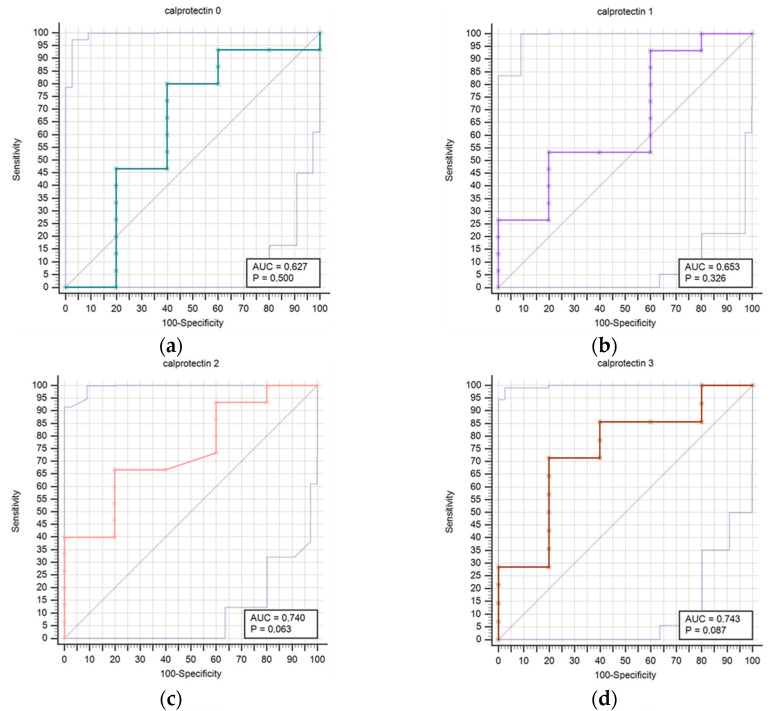

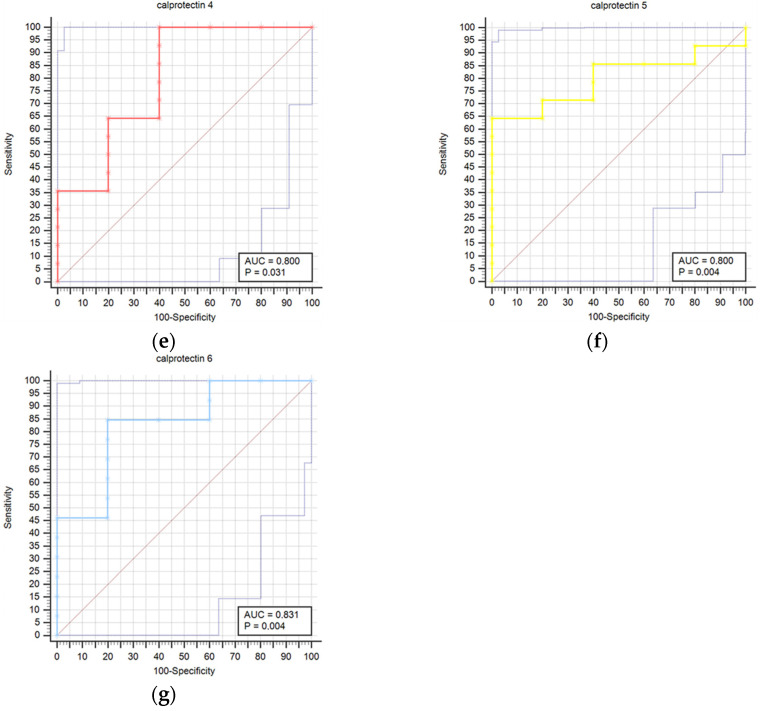
Receiver operating characteristic curves (ROC) comparison for outcome prediction based on the serum calprotectin concentrations at time points 0 (**a**), 1 (**b**), 2 (**c**), 3 (**d**), 4 (**e**), 5 (**f**), and 6 (**g**).

Figure 2 presents a comparison of receiver operating characteristic curves (ROC) for outcome prediction using serum calprotectin concentrations at time points T0 through T6. This visual depiction illustrates calprotectin’s prognostic accuracy at various stages of the study. The calprotectin concentrations were not statistically correlated with infectious complications at any time point.

Additionally, Spearman’s rank correlation confirmed that 90-day mRS was positively correlated with initial NIHSS (ρ = 0.75, *p* < 0.001) and infarct volume (ρ = 0.75, *p* < 0.001), while a negative correlation was found with PNI at the T0 time point (ρ = −0.86, *p* < 0.001). The Mann–Whitney test showed significant differences between the groups of survivors and deceased in PNI at the T0 time point (*p* = 0.010), initial NIHSS (*p* = 0.003), and infarct volume (*p* = 0.004). 

## 4. Discussion

Our results confirmed that higher concentrations of ST2 are associated with a worse 90-day outcome in IS. A statistically significant difference in ST2 concentrations was found between good and poor outcomes for all time points except T6. The difference in ST2 concentrations was also found between survivors and deceased for all time points except T0. Prognostic accuracy was highest for ST2 at T1 for a cut-off > 18.9 µg/L. The results also showed a statistically significant difference in calprotectin concentrations between good and poor outcomes at the T6 time point and between survivors and the deceased at the T5 time point. Prognostic accuracy was highest for calprotectin at the T5 time point for a cut-off > 4.5 mg/L.

The dynamics of ST2 and calprotectin at successive 24 h intervals over the first week following the beginning of IS symptoms are being examined for the first time in this study. Determining the ideal time point for biomarker assessment requires an understanding of the dynamics of a particular biomarker release. In order to ascertain the biomarkers with the greatest prognostic accuracy, we conducted measurements of biomarker concentrations at consecutive time points.

Consistent with our findings, prior research on humans has indicated that increased levels of soluble ST2 (sST2) serve as a prognostic indicator for the outcome of ischemic stroke. This further supports the potential usefulness of sST2 as a prediction tool for evaluating stroke prognosis [14,29,30,31,32]. The methodology applied in studies investigating the role of the ST2 biomarker in IS demonstrates variability, particularly with regard to study design, patient demographics, sample size, biomarker measuring methodologies, and outcome measures. Certain studies may concentrate on particular subgroups of individuals with ischemic stroke, such as those who have specific comorbidities or risk factors, while others may adopt more comprehensive inclusion criteria. Furthermore, disparities in the timing of biomarker assessments in relation to the beginning of stroke, together with variances in the duration of follow-up and the methodologies used to assess outcomes, can lead to methodological disparities among these studies. Additionally, the selection of statistical analysis methods and adjustments for possible confounding variables may differ, which might affect how findings from various research are interpreted and comparable.

In the discussion that follows, we list the studies in order of the number of measurements. It is crucial to note that none of these studies measured consecutively during the first week after the onset of IS symptoms. Most of these studies measured the concentration of biomarkers at a single time point, but some also measured at multiple points, up to a maximum of five. 

According to the literature data, we identified three studies that measured ST2 levels at one time point after IS. The first translational study, which demonstrated that the levels of the inhibitory IL-33 receptor sST2 are increased in human stroke patients, additionally measured ST2 levels in 53 IS patients with a middle cerebral artery non-lacunar stroke during the first 4.5 h of symptom onset. All patients received rt-PA (alteplase). This study showed increased levels of sST2 in the stroke patient plasma compared to healthy controls in the early course of stroke development. When a ROC curve was used to determine the cut point for ST2, it was shown that significantly more patients than controls had high levels of ST2. Plasma ST2 levels were higher in IS patients who were dependent 3 months after the IS (mRS > 2) than in stroke patients who recovered [33]. Two other studies were conducted on the Chinese population [29,30]. In the first one, ST2 concentrations were examined within 48 h of the onset of the first IS in 112 patients receiving thrombolysis therapy. Compared to patients without IS in the control group, patients with IS had significantly increased serum sST2 levels, significantly higher in the severe IS group (NIHSS ≥6) than in the mild IS group. Additionally, the infarct volume and the degree of IS severity determined by the NIHSS were associated with increased serum sST2 levels [29]. These findings are consistent with ours, which showed significant differences in initial NIHSS and infarct volume between survivors and deceased groups. The second Chinese study showed that increased sST2 levels within 24 h of TIA/IS onset in 420 patients were associated with increased risks of poor outcome (combination of a new stroke event and all-cause death) within 90 days and 1 year [30]. In this study, the median time to blood sampling was 19 h, compared to the median of our T0, which was 14 h, and the median of the NIHSS was two, which was significantly lower than our 13. ST2 has been analyzed more than once in the studies discussed further. In the study of Wolcott et al., sST2 was measured twice: at baseline (7.1 ± 3.3 h) in 664 patients and 48 h after stroke onset in 210 patients. At both time points, sST2 was confirmed as an independent predictor of 90-day mortality, regardless of the underlying cardiovascular disease. Additionally, sST2 predicted the subsequent development of hemorrhagic transformation in IS [14]. In an Indian study, plasma samples were evaluated at three time points within 24 h of IS onset in 108 patients (median NIHSS 12). Elevated levels of sST2, measured at 24 h from IS onset, were found to be an independent predictor of poor functional outcome at 3 months. The odds ratio was 6.44 (95% confidence interval: 1.40–46.3, *p* = 0.029) when using a cut-off value of 71.8 ng/mL. The specificity of this prediction was 96.9%, while the sensitivity was 38.5%. Applying a cut-off value of sST2 levels at 24 h to the model increased the probability of an unfavorable outcome by six times. This resulted in improved discriminative capacity and high specificity [32]. The dynamics of the increase in sST2 concentration within 24 h and the rising prognostic accuracy for the outcome are consistent with those in our investigation. However, a direct comparison of the findings is not feasible, given that the sampling in this study was completed within 24 h. In the French study, the level of sST2 in patient sera was evaluated at five time points (admission, 6, 24, 48 h, and 3 months) for 152 IS patients treated with MT. sST2 peaked at 6 h, and a high level of sST2 at 24 h was associated with all-cause death within the first 12 months [31]. In our study, ST2 peaked in T1 (median 38 h), likely due to a different disease course when no intervention was undertaken. Despite methodological differences, all of these studies produced similar results, but none provided a precise time point for measuring the biomarker concentration.

Contrary to our and the previously mentioned studies, the Linz Stroke Unit Study’s large cohort of acute IS patients did not confirm sST2 at admission as a strong and independent prognostic biomarker for 90-day all-cause mortality, with a median baseline NIHSS of 3 and a median time from stroke onset to blood draw of 13 h. However, the baseline plasma concentration was significantly higher among decedents from all causes than in survivors (AUC, 0.73; 95% CI, 0.69–0.76) [3].

In animal models employing models of IS, the findings support the notion that IL-33/ST2 signal transduction activates protective immune responses, thereby preventing the deterioration of ischemic brain injury [33,34,35]. Sastre et al. developed a model for ST2/IL-33 in CNS injury, which summarizes the phases of inflammation. The acute inflammatory response is marked by the release of danger-associated molecular patterns (DAMPs), the activation of local microglia, the infiltration of peripheral leukocytes, the release of pro-inflammatory cytokines and chemokines, the production of reactive oxygen species, and the release of matrix metalloproteases. These occurrences lead to a disruption of the blood–brain barrier, the development of brain edema, and secondary brain damage mediated by inflammation. A phase of immune-mediated repair occurs when the post-ischemic inflammation peaks about 3–4 days after an IS. The pro-inflammatory response may be amplified during the amplification phase by high sST2 levels, which may hinder IL-33’s neuroprotective action and instead impede IL-33 signaling. When sST2 levels decrease, IL-33 can interact with the ST2L receptor on macrophages and microglia to promote an anti-inflammatory response [13]. This concept and the role of ST2 in IS are supported by our results, which reveal high ST2 concentrations to the time point T3 (approximately within 86 h) and a consequent decrease in concentrations.

Our study design of calprotectin dynamics reveals an association with IS outcome after the T4 time point (median 110 h). This finding exhibits a modest deviation from prior research, which assessed calprotectin levels solely at a single time point during the earlier stages of the disease [18,19,20]. The Chinese multicenter prospective study among 4785 patients with IS from two independent cohorts measured calprotectin concentrations, showing that baseline concentrations (within 72 h) were independently associated with increased risks of adverse clinical outcomes at 3 months following IS [18]. Another Chinese study that enrolled 271 patients found significantly higher plasma calprotectin concentrations in patients with IS compared to 145 healthy non-stroke controls. Furthermore, the study observed significantly higher calprotectin concentrations in patients with a poor prognosis compared to those with a good prognosis. Furthermore, the study demonstrated an independent relationship between baseline calprotectin levels within 24 h and the progression of IS disease during a 2-week follow-up interval [19]. In a Spanish study, higher calprotectin levels were associated with 3-month mortality, hemorrhagic transformation, and lower 3-month functional independence in 748 patients sampled within the first 24 h after IS onset [20]. In all these studies, calprotectin was analyzed only once, and the different study designs could explain the discrepancy in the results. According to the Korean study, 27 stroke patients had significantly higher levels of fecal calprotectin in their feces than in the controls. This finding raises the possibility that elevated fecal calprotectin is linked to consciousness and systemic response in stroke patients [36].

Our study’s advantages included thorough sample collection at predetermined intervals following the onset of IS. Instead of taking biomarker measurements hours after admission, we determined biomarker concentrations at consecutive time points (24 h intervals) within the first week after IS onset to identify time points with the highest prognostic accuracy. The homogeneity of our sample is enhanced by the inclusion of only patients with moderate to severe IS (NIHSS ≥ 8). Additionally, patients with IS who had not received thrombolysis or MT were included in the study because we intended to investigate how the disease developed naturally without the potential of influencing the concentration of serum biomarkers. Based on our and previously described studies’ results, monitoring ST2 might, at a specific time point, be considered a biomarker for the prognosis of IS, like the current ACC/AHA guideline for heart failure management [37]. Additionally, the results of our study related to ST2 concentrations support a previously proposed model with the ST2/IL-33 system regulating the acute immune response, suggesting that therapeutic intervention focused on components of the ST2/IL-33 system may be considered a novel approach for IS [32,38].

There are several limitations to this study. The generalizability of our findings could be constrained by the monocentric design and the small sample size. Given that the study included moderate to severe strokes (NIHSS ≥ 8), our findings might not apply to all acute stroke patients in the emergency room, potentially introducing bias into our results due to selection. Therefore, caution should be exercised when extrapolating these findings to broader populations or clinical settings.

## 5. Conclusions

The study’s results showed that although calprotectin did not fully meet expectations for outcome and survival, it was useful for predicting values at later measurement time points. However, when looking at IS functional outcome and mortality, ST2 was found to be an important biomarker with strong predictive value across all measurement time points.

The study’s findings have established the role of the ST2 dynamic in the outcome of IS, opening up avenues for future research with a larger sample to explore its potential for clinical application. This potential is particularly promising in the early stages and immediately following the onset of IS, where the use of ST2 as a biomarker could significantly impact patient care. 

## Figures and Tables

**Table 1:** The procedure manual and breakdown of study time points in relation to time, blood sampling, and clinical and neurological indices.

Time Point	Relates to Time	Clinical Neurological Indices
**T0**	Immediately upon receipt at the Department of Neurology, recording the exact time of onset of symptoms	Medical history; Risk factors; Comorbidities; NIHSS; Blood sampling; PNI; GPS; Brain CT
**T1**	At 7:30 a.m. the first morning after admission to the hospital	Comorbidities; Complications; NIHSS; Blood sampling; PNI; GPS; Control brain CT (at an interval of at least 20–24 h from the initial one); CT volumetry
**T2**	At 7:30 a.m. the second morning after admission to the hospital	Comorbidities; Complications; NIHSS; Blood sampling; PNI; GPS
**T3**	At 7:30 a.m. the third morning after admission to the hospital	Comorbidities; Complications; NIHSS; Blood sampling; PNI; GPS
**T4**	At 7:30 a.m. the fourth morning after admission to the hospital	Comorbidities; Complications; NIHSS; Blood sampling; PNI; GPS
**T5**	At 7:30 a.m. the fifth morning after admission to the hospital	Comorbidities; Complications; NIHSS; Blood sampling; PNI; GPS
**T6**	At 7:30 a.m. the sixth morning after admission to the hospital	Comorbidities; Complications; NIHSS; Blood sampling; PNI; GPS; TOAST; OCSP; mRS
**T7**	90th day (±7 days) after admission to the hospital	Comorbidities; Complications; NIHSS; mRS

T0—time point 0. T1—time point 1. T2—time point 2. T3—time point 3. T4—time point 4. T5—time point 5. T6—time point 6. T7—time point 7. NIHSS—National Institutes of Health Stroke Scale. PNI—Prognostic nutritional index. GPS—Glasgow Prognostic Score. CT—computed tomography. TOAST—Trial of Org 10172 in Acute Stroke Treatment. OCSP—Oxfordshire Community Stroke Project. mRS—modified Rankin score.

**Table 2:** Overview of baseline demographic information, clinical parameters, and relevant medical history of patients with ischemic stroke (*n* = 20).

Baseline Demographic Information, Clinical Parameters, and Relevant Medical History of Patients with Ischemic (Expressed in the Suitable Measurement)	
Female sex, *n* (%)	13 (65%)
Age, years—median (IQR)	84 (46–94)
Body mass index, kg/m^2^—median (IQR)	24.5 (23.7–29.3)
Active smoking, *n* (%)	5 (25%)
Alcohol abuse, *n* (%)	4 (20%)
Psychotropic substances, *n*	0
Arterial hypertension, *n* (%)	18 (90%)
Atrial fibrillation, *n* (%)	9 (45%)
Presence of an artificial heart valve, *n* (%)	1 (5%)
Dyslipidaemia, *n* (%)	9 (45%)
Diabetes mellitus, *n* (%)	3 (15%)
Coronary heart disease, *n* (%)	1 (5%)
Peripheral arterial disease, *n* (%)	1 (5%)
Prior stroke/TIA, *n* (%)	4 (20%)
Average time from onset to sampling, hours—median (IQR)	14 (6–16)
NIHSS at admission—median (IQR)	13 (8–17)
Infarct volume, cm^2^—median (IQR)	12.6 (1.6–105.7)
**Stroke etiology, TOAST subtype**	
Large-artery atherosclerosis, *n* (%)	4 (20%)
Small-vessel occlusion (lacune), *n* (%)	9 (45%)
Cardioembolism, *n* (%)	7 (35%)
**Stroke syndrome, OCSP classification**	
TACI, *n* (%)	1 (5%)
PACI, *n* (%)	13 (65%)
LACI, *n* (%)	4 (20%)
POCI, *n* (%)	2 (10%)

IQR—interquartile range. TIA—transient ischemic attack. NIHSS—National Institutes of Health Stroke Scale. TOAST—Trial of Org 10172 in Acute Stroke Treatment. OCSP—Oxfordshire Community Stroke Project. TACI—total anterior circulation infarct. PACI—partial anterior circulation infarct. LACI—lacunar infarct. POCI—posterior circulation infarct.

**Table 3:** The 90-day primary (functional) and secondary (all-cause mortality) outcome in patients with ischemic stroke (*n* = 20). The primary outcome was defined as 0–2 for a good outcome and 3–6 for a poor outcome.

The 90-Day Primary and Secondary Outcome in Patients with Ischemic Stroke	*n* (%)
mRS 0–2, n (%)	5 (25%)
mRS 3–6, *n* (%)	15 (75%)
All-cause mortality, *n* (%)	8 (40%)

mRS—modified Rankin score.

**Table 4:** The median concentrations of ST2 and calprotectin at the measurement time points T0–T6 expressed in median hours.

Variable	T0	T1	T2	T3	T4	T5	T6
**Measurement time points, hours—median** **(IQR)**	14 (6–16)	38 (30–40)	62 (54–64)	86 (78–88)	110 (102–112)	134 (126–143)	158 (150–161)
**ST2 (µg /L),** **median (IQR)**	23.95 (17.00–46.45)	25.25 (15.15–42.05)	22.20 (14.50–37.60)	22.60 (13.48–32.18)	16.70 (11.93–37.30)	17.20 (10.78–38.75)	13.40 (10.30–23.10)
**Calprotectin (mg/L),** **median (IQR)**	5.86 (3.56–7.65)	5.04 (2.93–6.78)	3.86 (3.03–5.97)	4.85 (3.08–5.91)	4.06 (3.54–5.84)	4.47 (2.98–6.88)	4.89 (3.19–5.69)

IQR—interquartile range. T0—time point 0. T1—time point 1. T2—time point 2. T3—time point 3. T4—time point 4. T5—time point 5. T6—time point 6.

**Table 5:** Differences in ST2 concentrations, measured in ug/L, between good (modified Rankin score 0–2) and poor outcomes (modified Rankin score 3–6) at day 90 (+/− 7 days), expressed in median and interquartile range at each measurement time point T0–T6.

Time Point	Good Outcome ST2 Concentrations (µg /L), Median (IQR)	Poor Outcome ST2 Concentrations (µg /L), Median (IQR)	*p*-Value
**T0**	13.77 (11.03–23.49)	28.64 (19.51–59.46)	0.04
**T1**	10.75 (9.39–17.94)	28.59 (23.67–50.55)	0.006
**T2**	10.34 (7.81–17.29)	28.39 (16.78–58.27)	0.01
**T3**	9.02 (8.11–17.88)	26.24 (14.79–38.66)	0.021
**T4**	11.30 (8.33–13.73)	28.37 (16.16–41.53)	0.007
**T5**	9.70 (7.73–15.18)	21.29 (14.56–43.36)	0.033
**T6**	10.59 (8.08–14.26)	15.23 (12.31–52.06)	0.073

IQR—interquartile range. T0—time point 0. T1—time point 1. T2—time point 2. T3—time point 3. T4—time point 4. T5—time point 5. T6—time point 6.

**Table 6:** Differences in ST2 concentrations, measured in µg /L, between survivors (modified Rankin score 0–5) and poor outcome (modified Rankin score 6) at day 90 (± 7 days), expressed in median and interquartile range at each measurement time point T0–T6.

Time Point	Survivors ST2 Concentrations (µg /L), Median (IQR)	Deceased ST2 Concentrations (µg /L), Median (IQR)	*p*-Value
**T0**	20.51 (14.85 –27.12)	40.91 (23.43–68.30)	0.064
**T1**	18.25 (12.95–26.65)	47.40 (27.16–66.67)	0.014
**T2**	16.27 (12.02–23.26)	53.13 (26.04–66.67)	0.009
**T3**	14.56 (10.70–23.68)	38.66 (30.31–69.80)	0.003
**T4**	14.82 (10.48–17.62)	41.53 (36.70–55.31)	0.004
**T5**	13.78 (9.67 –19.19)	43.36 (35.83–83.18)	0.004
**T6**	12.90 (10.45–15.23)	66.02 (31.16–79.98)	0.027

IQR—interquartile range. T0—time point 0. T1—time point 1. T2—time point 2. T3—time point 3. T4—time point 4. T5—time point 5. T6—time point 6.

**Table 7:** Prognostic accuracy of ST2 concentrations at time points (T0–T6).

Time Point	Cut-Off (µg/L)	Sensitivity (95%Cl)	Specificity (95%Cl)	PPV (95%Cl)	NPV (95%Cl)	LR+ (95%Cl)	LR− (95%Cl)	AUC
**T0**	>13.8	93.3 (68.1–99.8)	60.0 (14.7–94.7)	87.5 (70.3–95.4)	75.0 (28.4–95.8)	2.3 (0.8–6.9)	0.1 (0.02–0.8)	0.007
**T1**	>18.9	80.0 (51.9–95.7)	100.0 (47.8–100.0)	100.0 (/)	62.5 (37.7–82.1)	/	0.2 (0.07–0.6)	<0.0001
**T2**	>18	73.3 (44.9–92.2)	100.0 (47.8–100.0)	100.0 (/)	55.6 (35.1–74.3)	/	0.3 (0.1–0.6)	<0.0001
**T3**	>13.2	92.9 (66.1–99.8)	80.0 (28.4–99.5)	92.9 (69.1–96.7)	80.0 (36.5–96.5)	4.6 (0.8–26.9)	0.09 (0.1–0.6)	0.006
**T4**	>15.3	78.57 (49.2–95.3)	100.0 (47.8–100.0)	100.0 (/)	62.6 (37.9–82.0)	/	0.2 (0.08–0.6)	<0.0001
**T5**	>13	78.6 (49.3–95.3)	80.0 (28.4–99.5)	91.7 (65.1–98.5)	57.1 (30.9–79.9)	3.9 (0.7–23.2)	0.3 (0.1–0.8)	0.006
**T6**	>13	61.5 (31.6–86.1)	80.0 (28.4–99.5)	88.9 (56.9–96.0)	44.4 (26.1–64.4)	3.1 (0.5–18.7)	0.5 (0.2–1.1)	0.09

CI—confidence interval. PPV—positive predictive value. NPV—negative predictive value. LR+—positive likelihood ratio. LR−—negative likelihood ratio. AUC—area under the curve.

**Table 8:** Differences in calprotectin concentrations, measured in mg/L, between good (modified Rankin score 0–2) and poor outcomes (modified Rankin score 3–6) at day 90 (± 7 days), expressed in median and interquartile range at each measurement time point T0–T6.

Time Point	Good Outcome Calprotectin Concentrations (mg/L), Median (IQR)	Poor Outcome Calprotectin Concentrations (µg /L), Median (IQR)	*p*-Value
**T0**	7.44 (4.48–8.68)	5.84 (3.47–7.15)	0.407
**T1**	4.68 (1.80–5.80)	5.42 (3.09–7.89)	0.316
**T2**	3.27 (1.13–3.47)	3.93 (3.16–6.52)	0.106
**T3**	3.03 (2.17–4.42)	5.35 (3.50–7.11)	0.116
**T4**	2.75 (2.40–4.20)	4.44 (3.61–6.39)	0.052
**T5**	2.97 (1.98–3.81)	5.60 (3.07–7.18)	0.052
**T6**	3.19 (1.96–4.00)	5.15 (4.29–5.96)	0.034

IQR—interquartile range. T0—time point 0. T1—time point 1. T2—time point 2. T3—time point 3. T4—time point 4. T5—time point 5. T6—time point 6.

**Table 9:** Differences in calprotectin concentrations, measured in mg/L, between survivors (modified Rankin score 0–5) and poor outcome (modified Rankin score 6) at day 90 (±7 days), expressed in median and interquartile range at each measurement time point T0–T6.

Time Point	Survivors Calprotectin Concentrations (mg/L), Median (IQR)	Deceased Calprotectin Concentrations (mg/L), Median (IQR)	*p*-Value
**T0**	5.55 (3.56–7.64)	6.06 (3.92–7.95)	0.643
**T1**	4.08 (2.92–6.33)	5.99 (3.10–9.24)	0.355
**T2**	3.90 (3.28–4.71)	3.19 (2.36–7.51)	0.699
**T3**	4.10 (2.80–5.89)	5.08 (3.64–7.39)	0.447
**T4**	3.98 (3.18–4.87)	6.09 (3.54–6.45)	0.272
**T5**	3.33 (1.93–4.57)	7.18 (6.35–9.78)	0.004
**T6**	4.05 (2.95–5.26)	5.67 (5.03–5.92)	0.160

IQR—interquartile range. T0—time point 0. T1—time point 1. T2—time point 2. T3—time point 3. T4—time point 4. T5—time point 5. T6—time point 6.

**Table 10:** Prognostic accuracy of calprotectin concentrations at time points (T0–T6).

Time Point	Cut-Off (mg/L)	Sensitivity (95%Cl)	Specificity (95%Cl)	PPV (95%Cl)	NPV (95%Cl)	LR+ (95%Cl)	LR− (95%Cl)	AUC
**T0**	≤7.4	80.0 (51.9–95.7)	60.0 (14.7–94.7)	85.7 (66.6–94.8)	50.0 (22.7–77.5)	2.0 (0.7–6.0)	0.3 (0.1–1.2)	0.500
**T1**	>2.2	93.3 (68.1–99.8)	40.0 (5.3–85.3)	82.5 (69.3–90.6)	66.7 (18.5–94.6)	1.6 (0.8–3.2)	0.2 (0.02–1.6)	0.326
**T2**	>3.3	66.7 (38.4–88.2)	80.0 (47.8–100.0)	83.3 (61.7–93.9)	37.5 (17.9–62.3)	3.3 (0.6–20.0)	0.4 (0.2–1.0)	0.063
**T3**	>3.9	71.4 (41.9–91.6)	80.0 (28.4–99.5)	90.9 (62.7–98.3)	50.0 (28.1–71.9)	3.6 (0.6–21.3)	0.4 (0.1–0.9)	0.087
**T4**	>2.8	100.0 (76.8–100.0)	60.0 (14.7–94.7)	87.5 (70.5–95.3)	100 (/)	2.5 (0.9–7.3)	/	0.031
**T5**	>4.5	64.3 (35.1–87.2)	100.0 (47.8–100.0)	100 (/)	50.0 (33.1–66.9)	/	0.4 (0.2–0.7)	0.004
**T6**	>3.6	84.6 (54.6–98.1)	80.0 (28.4–99.5)	91.7 (65.2–98.5)	57.1 (31.1–79.8)	3.9 (0.7–22.8)	0.3 (0.1–0.9)	0.004

Cl—confidence interval. PPV—positive predictive value. NPV—negative predictive value. LR+—positive likelihood ratio. LR−—negative likelihood ratio. AUC—area under the curve.

## Data Availability

The data that support the findings of this study are available on request from the corresponding author. The data are not publicly available due to privacy or ethical restrictions.
